# Availability of exercise facilities and physical activity in 2,037 adults: cross-sectional results from the Swedish neighborhood and physical activity (SNAP) study

**DOI:** 10.1186/1471-2458-12-607

**Published:** 2012-08-03

**Authors:** Ulf Eriksson, Daniel Arvidsson, Kristina Sundquist

**Affiliations:** 1 Center for Primary Health Care Research, Lund University, Skåne University Hospital, CRC, Building 28, Floor 11, Entrance 72, S-205 02, Malmö, Sweden

## Abstract

**Background:**

Exercise facilities may have the potential to promote physical activity among residents, and to support an active lifestyle throughout the year. We investigated the association between objectively assessed availability of exercise facilities and objectively assessed physical activity outcomes, and whether time of year had a modifying effect on these associations.

**Methods:**

A total of 2,037 adults (55% females) wore an accelerometer for seven days. Time spent in moderate to vigorous physical activity (minutes per day) and meeting the physical activity recommendations (yes/no) were used as outcome variables. Availability of exercise facilities was measured within 1,000-meter line-based road network buffers around participants’ residential addresses using Geographic Information Systems. Socio-demographic variables and time of year were included as covariates in the analyses.

**Results:**

Participants with ≥4 exercise facilities within their buffer zones spent 5.4 (confidence interval (CI) = 2.3-8.5) more minutes in moderate to vigorous physical activity per day, and had 69% higher odds (OR = 1.69; CI = 1.39-2.05) of meeting the physical activity recommendations, compared to those with no exercise facilities within their buffer zones. Time of year had no modifying effect on these associations.

**Conclusions:**

Our results show that objective availability of exercise facilities was associated with accelerometer-assessed time spent in moderate to vigorous physical activity and the odds of meeting the recommended levels of physical activity. Neighborhoods may be a logical and potentially significant venue for policy interventions aimed at increasing physical activity in the overall population.

## Background

Although physical activity is known to influence human health [1-3], large proportions of populations worldwide do not meet recommended levels of physical activity [4,5]. According to the World Health Organization, insufficient levels of physical activity are one of the top contributors to global mortality [6]. It is therefore a highly important public health priority to increase the proportion of physically active people.

Interventions aimed at increasing levels of physical activity have, however, had varying success [7,8]. Recently, considerable efforts have been made to implement ecological models for physical activity behavior. These ecological models often include attributes of the built neighborhood environment [9-11].

Specific attributes of the built neighborhood environment that may have the potential to promote physical activity among residents include neighborhood availability of exercise facilities. Studies examining the association between availability of exercise facilities and physical activity have, however, produced varying results. A review from 2008 found little or no evidence for an association between availability of physical activity facilities and walking for transportation or recreational walking [12]. In contrast, a study from the U.S. found an association between density of exercise facilities and exercise prevalence in study participants from three areas with widely varying population densities [13]. This association was modified by income and race/ethnicity, being stronger among those with low incomes and non-Hispanic Black and Hispanic participants compared to their high-income and non-Hispanic White counterparts. Income was also found to be an effect modifier in another study, which detected an association between number of gyms per square kilometer and physical activity in low-income women but not high-income women [14]. Hence, associations between exercise facilities and physical activity may be influenced by individual characteristics. If this is the case, it is possible that neighborhood characteristics aimed at increasing people’s physical activity may not reach all population groups to an equal extent.

A majority of previous studies were based on self-reported physical activity and/or self-reported neighborhood availability of exercise facilities. Same-source bias may generate spurious associations if the neighborhood characteristic and the outcome are collected by self-report, as different variables collected from the same source may not be independent from each other. In addition, self-reported measures of physical activity are often biased by over-reporting, social desirability and other factors [15]. These types of biases can be avoided if physical activity is measured objectively, for example by accelerometry.

Different methods exist to objectively assess the availability of exercise facilities, and the choice of method may influence the accuracy of neighborhood assessments. A crude method of objectively measuring availability of exercise facilities is to assess neighborhood availability of exercise facilities within administrative areas, such as census tracts or provinces [14,16,17]. All residents living within these administrative areas are considered to have the same availability of exercise facilities. To obtain a more individualized measure of neighborhood availability of exercise facilities, a buffer zone may be created around each individual’s residential address [13,14,18]. Circular buffer zones are easy to create but may include areas that are not accessible to participants due to, for example, rivers and other natural and unnatural barriers. Buffer zones based on the road network may provide a more accurate picture of the neighborhood facilities that are actually available to residents [19].

The present Swedish study represents a novel contribution because both the predictor variable (neighborhood availability of exercise facilities) and the outcome variable (physical activity) were measured objectively. Moreover, Sweden is particularly well suited for this kind of study due to its temperate climate. In countries with temperate climates, where the four seasons are well defined, time of year may have an impact on people’s physical activity. Previous studies have shown an association between time of year and physical activity, with lower levels of physical activity occurring during winter [20-22]. It has been hypothesized that exercise facilities could be of importance in supporting a physically active lifestyle throughout the year [23]. This suggests a stronger association between availability of indoor exercise facilities and physical activity during the winter than during the summer. To our knowledge, no previous study using objective measures of availability of exercise facilities and physical activity has explored this hypothesis.

The main aim of this study was to investigate the association between objective availability of exercise facilities, measured within line-based road network buffer zones around participants’ residences, and objectively assessed physical activity outcomes. We also aimed to investigate the possible effect of socio-demographic variables and time of year on this association (effect modification).

## Methods

### Design and study sample

The data used in this cross-sectional study were collected between November 2008 and November 2009 in Stockholm, Sweden as part of the Swedish Neighborhood and Physical Activity (SNAP) study. The SNAP study was originally designed to investigate the association between neighborhood walkability and physical activity [24]. A total of 32 neighborhoods were sampled based on walkability (based on data provided by Statistics Sweden, the City Planning Administration in Stockholm and the company Teleadress) and neighborhood income (based on data provided by Statistics Sweden) in order to ensure variation in neighborhood-level walkability and socio-economic status. Data were collected throughout the study period, except between 9 December 2008 and 12 January 2009 and between 16 June and 17 August 2009 (these two time periods correspond to the winter and summer holidays in Sweden, respectively).

The sampling procedure has been described in detail elsewhere [24]. Briefly, neighborhood walkability and income were calculated for all 408 basic areas (neighborhoods) in the city of Stockholm. Geographic Information Systems (GIS) were used to calculate walkability as an index comprising z-scores for residential density, street connectivity and land use mix. Neighborhoods in the first to fourth walkability index deciles were considered less walkable, and those in the seventh to tenth deciles where considered highly walkable. Neighborhood income in each area was calculated as the median disposable family income, taking the number and age of family members into account. Neighborhoods in the second to fourth neighborhood income deciles were considered to be of low income, and those in the seventh to ninth deciles of high income. Four neighborhood categories were created: high walkability/high income, high walkability/low income, low walkability/high income and low walkability/low income. A total of 32 neighborhoods (eight from each category) were sampled for the study.

The SNAP study aimed to recruit 75 participants from each of the 32 neighborhoods, i.e. 2,400 in total. Simple random sampling of 8,000 individuals aged 20 to 65 (200 from each neighborhood) was performed by the Stockholm Office of Research and Statistics. Immigrants who had arrived in Sweden after 2003 were excluded since knowledge of Swedish was an inclusion criterion (see below). A total of 6,089 individuals had a listed landline or mobile phone number and were included in the recruitment procedure. Of the 4,747 individuals who were reached, 4,369 met the three inclusion criteria: (1) being able to read and write Swedish, (2) having lived in the neighborhood for at least three months, and (3) having no serious impaired ability to walk. The final study population for analyses, after exclusion due to missing data, consisted of 2,037 individuals, which gave a response rate of 47% (2,037/4,369). Recruitment of participants was performed concurrently in all included neighborhoods by the telemarketing company Markör AB (Örebro, Sweden). Markör AB has previously been involved in the recruitment of participants for large-scale research studies. Lists of enrolled participants were delivered to us on a weekly basis and a package containing an accelerometer, an accelerometer logbook, a questionnaire and a prepaid return envelope was sent to the residential address of each participant.

### Availability of exercise facilities

**Figure 1:**
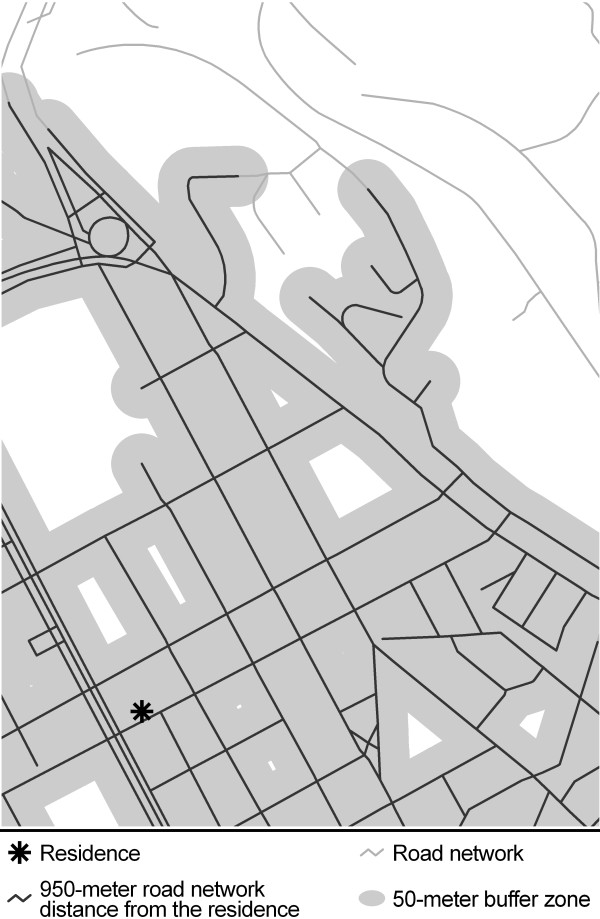
**Line-based network buffer zone.** Example of a line-based network buffer zone (950 + 50 meters).

Availability of exercise facilities was objectively measured using GIS. To assess area of exposure, neighborhoods were defined by creating a buffer zone originating from the residential address of each participant using the Network Analyst extension in ArcGIS/ArcInfo 9.2 (ESRI Inc., Redlands, California, USA). Data on the road network, including cycle paths and footpaths, was obtained from the City Planning Administration in Stockholm. Line-based network buffer zones were created by following the road network in all possible directions from each residence for 950 meters, and then creating a 50-meter buffer zone in all directions from the center of the road (Figure 1). 1,000-meter buffer zones are likely to represent areas that can be reached in daily life by a large majority of the adult population and have been used to define neighborhoods in previous research [25,26]. Data from 2008 on the locations and business names of exercise facilities were provided by Teleadress, a company created when the government-owned telecoms agency was privatized and one of the leading providers of geo-coded data on businesses and private individuals in Sweden. The data from Teleadress included privately and publicly owned exercise facilities that have a registered telephone number and/or those that had provided information about their existence to Teleadress. The database is updated continuously and inclusion is free of charge. The data included nine categories of exercise facilities: “gym/fitness center”, “sport facility”, “tennis court”, “dance class center”, “public ice rink”, “squash court”, “sports hall”, “public baths” and “badminton court”. Most facilities were indoor facilities; only a few in the category “tennis court” were outdoor facilities. A vast majority of the exercise facilities were charged. Exercise facilities located within buffer zones were manually screened to identify those that did not offer exercise to the adult population. These facilities, as well as those not offering any exercise opportunities on site, were excluded. We identified 341 exercise facilities; 58 of these were excluded because they did not offer exercise to the adult population on site. Individual exercise facilities offering more than one activity received a count for each activity. For example, an exercise facility listed in both the “gym/fitness center” and “squash court” categories was counted as two facilities. The category “sport facility” was often present as a general description together with a more specific category. For example, gyms often appeared in both the “sport facility” and “gym/fitness center” categories. “Sport facility” was thus only counted when the only category present, and not when accompanied by another exercise facility category.

### Time spent in moderate to vigorous physical activity

Actigraph GT1M accelerometers (ActiGraph, Pensacola, Florida, USA) were used to objective measure participants’ physical activity. Participants were asked to wear the accelerometer on the hip or lower back for 7 consecutive days and to remove it only when sleeping or engaging in water-based activities. A study comparing placement of accelerometers on the hip or lower back under free-living conditions found that the position of the accelerometer had no effect on the estimation of time spent in moderate to vigorous physical activity [27]. Four standardized text messages were sent to each participant’s cell phone during the 7-day measurement period to improve compliance. The Actigraph GT1M measures acceleration in the vertical axis at a frequency of 30 times per second (30 Hertz). These accelerations are summed within 60-second periods (epoch) and the output is referred to as “counts”. Non-wear time was defined as 30 or more consecutive minutes with zero counts, and 10 h of wear time was required to constitute a valid day. Accelerometer wear time was calculated by subtracting non-wear time from 24 h. Variance analysis of our own accelerometer data showed that 6 or 7 valid days were required for inclusion in the analysis [28]. Time spent in moderate to vigorous physical activity was determined using Freedson’s cut-off point for accelerometer counts [29], which is ≥1,952 counts/min. This cut-off was applied to each minute of wear time for the valid days. The mean time per day spent in moderate to vigorous physical activity during all valid days was used as the outcome variable.

### Meeting physical activity recommendations

According to the global physical activity recommendations of the World Health Organization, adults should engage in ≥150 min of moderate physical activity or ≥75 minutes of vigorous physical activity per week, or an equivalent combination of the two. Activities should be performed in bouts of ≥10 min [30]. In the present study, participants were considered to have met these recommendations if they accumulated ≥150 min of moderate to vigorous physical activity in bouts of ≥10 min within a week. Bouts of moderate to vigorous physical activity were identified as 10 or more consecutive minutes with ≥1,952 counts per minute. During each bout of physical activity, the number of counts per minute was permitted to dip below this cut-off for 1-2 min. This approach, which allows for brief pauses in physical activity (for example when stopping at a red light or tying a shoelace), is recommended [31] and has been used previously [5]. Bouts of physical activity were identified during wear time on valid days as defined above. Weekly time spent in bouts of moderate to vigorous physical activity for participants with 6 valid days were extrapolated to 7 days using the mean of the six valid days (mean value for the 6 valid days multiplied by 7).

### Time of year

The year was divided into four periods: January-March, April-June, July-September and October-December. The Swedish climate offers substantial weather variation. According to the Swedish Meteorological and Hydrological Institute (http://www.smhi.se/en/services), daily mean air temperature varied between -7°C and +19°C in the city of Stockholm during the data collection period. January-March was the coldest period with a daily mean temperature of -1°C.

### Socio-demographic information

Participants’ socio-demographic information was based on self-report. Age was categorized as 20-30 years, 31-40 years, 41-50 years and 51-66 years. Marital status was dichotomized as married/cohabiting or single. Income was calculated by dividing the gross family income by number of people living in the household, with children under the age of 18 being given a consumption weight of 0.5. Income was then categorized as low (<150,000 SEK/year), middle (150,000-349,999 SEK/year) and high (≥350,000 SEK/year).

### Statistical analysis

The association between availability of exercise facilities and time spent in moderate to vigorous physical activity was analyzed by linear regression. Non-parametric cluster bootstrap estimates with 1,000 replications were applied due to the skewed distribution of the physical activity data. It is a method that constructs a number of resamples of the original dataset, each obtained by random replacements of the original dataset and assuming an identically distributed population. Bootstrapping techniques have been used in previous studies of the association between environmental attributes and physical activity [24,32]. Two models were created: a crude model including only availability of exercise facilities and physical activity, and a full model also including sex, age, income, marital status and time of year. The full model was also adjusted for accelerometer wear time since it was found to be a potential confounder (inclusion of this variable in the model resulted in a 10% change of the regression coefficients). Standard errors were corrected for clustering effects as the data were collected within 32 neighborhoods. The regression coefficients represent differences in minutes per day compared to the reference group. Interactions and multicollinearity between the explanatory variables in the full model were examined.

The association between availability of exercise facilities and whether or not participants met the physical activity recommendations (yes/no) was analyzed by logistic regression. Two models were created: a crude model including only availability of exercise facilities, and a full model also including sex, age, income, marital status and time of year. Accelerometer wear time was not a confounder and was not included in this model. Standard errors were corrected for clustering effects in the data. Interactions between explanatory variables in the full model were examined. Goodness of fit was estimated by the Hosmer-Lemeshow test [33].

All statistical analyses were performed using STATA 10.1 (StataCorp, College Station, Texas, USA) and statistical significance was determined at α < 0.05.

### Non-response analysis

Results from a telephone-based non-response analysis of 205 randomly selected non-responders showed that the proportion of females was slightly higher among participants compared to non-participants. Participants were slightly older than non-participants. There was no significant difference in income between participants and non-participants.

### Ethics

Ethical approval for this study was granted by the Ethics Committee of Karolinska Institutet, Stockholm. Written informed consent was obtained from all participants.

## Results

### General results

Descriptive statistics for the study participants are shown in Table 1. The overall median time spent in moderate to vigorous physical activity was 42 min per day (interquartile range = 28-58 min). The median time spent in moderate to vigorous physical activity among participants with 0, 1-3 and ≥4 exercise facilities within their buffer zones was 41, 41, and 47 min/day, respectively. The corresponding median time spent in 10-min bouts of moderate to vigorous physical activity was 14, 13 and 18 min/day, respectively. Overall, 35% of participants met the physical activity recommendation of ≥150 min of moderate to vigorous physical activity per week (31, 33 and 44% of participants with 0, 1-3, and ≥4 exercise facilities within their buffer zones, respectively). 55% of the participants were females; 77% were married/cohabiting. 57% were in the middle income group and 40% were over the age of 50.

**Table 1:** Descriptive statistics for the 2,037 individuals included in the study

		**Availability of exercise facilities**		
	**All**	**0**	**1-3**	**≥4**
		**n = 964**	**n = 626**	**n = 447**
	**Median (IQR)**	**Median (IQR)**	**Median (IQR)**	**Median (IQR)**
Moderate to vigorous physical activity (min/day)	42 (28-58)	41 (27-57)	41 (28-58)	47 (32-63)
Accelerometer wearing time (min/day)	861 (814-902)	862 (819-903)	863 (813-906)	855 (803-893)
	n (%)	n (%)	n (%)	n (%)
*Physical activity recommendations met*				
· Yes	704 (35)	303 (31)	205 (33)	196 (44)
· No	1333 (65)	661 (69)	421 (67)	251 (56)
*Gender*				
· Male	912 (45)	457 (47)	272 (43)	183 (41)
· Female	1125 (55)	507 (53)	354 (57)	264 (59)
*Age (years)*				
· 20–30	214 (11)	87 (9)	71 (11)	56 (13)
· 31–40	415 (20)	205 (21)	130 (21)	80 (18)
· 41–50	590 (29)	270 (28)	197 (31)	123 (28)
· 51–66	818 (40)	402 (42)	228 (36)	188 (42)
*Income*				
· Low	383 (19)	174 (18)	137 (22)	72 (16)
· Middle	1159 (57)	570 (59)	351 (56)	238 (53)
· High	495 (24)	220 (23)	138 (22)	137 (31)
*Marital status*				
· Married/cohabiting	1560 (77)	765 (79)	472 (75)	323 (72)
· Single	477 (23)	199 (21)	154 (25)	124 (28)
*Time of year*				
· January-March	576 (28)	254 (26)	194 (31)	128 (29)
· April-June	597 (29)	288 (30)	177 (28)	132 (30)
· July-September	257 (13)	136 (14)	73 (12)	48 (11)
· October-December	607 (30)	286 (30)	182 (29)	139 (31)

IQR: Interquartile range.

### Time spent in moderate to vigorous physical activity

Results from the crude linear regression model (Table 2, model A) show that participants with ≥4 exercise facilities within their buffer zones spent 5.4 more minutes per day in moderate to vigorous physical activity than those with no exercise facilities within their buffer zones (regression coefficient = 5.4, CI = 2.2-8.5). This difference remained statistically significant when sex, age, income, marital status, time of year and accelerometer wear time were included in the model (Table 2, model B). There was no significant difference in time spent in moderate to vigorous physical activity between participants with 1-3 exercise facilities within their buffer zones and those with no facilities. Single participants spent more time in moderate to vigorous physical activity than their married/cohabiting counterparts and participants aged 20-30 spent more time in moderate to vigorous physical activity than those over the age of 30. Neither time of year nor any of the other explanatory variables modified the association between availability of exercise facilities and time spent in moderate to vigorous physical activity (i.e., there was no effect modification).

**Table 2:** Linear regression analysis of predictors of moderate to vigorous physical activity

	**Model A**^**1**^	**Model B**^**2**^
*Availability of exercise facilities*		
· 0	Reference	Reference
· 1-3	0.5 (-1.4–2.4)	0.3 (-1.5–2.1)
· ≥4	5.4* (2.2–8.5)	5.4* (2.3–8.5)
*Gender*		
· Male		Reference
· Female		-2.4 (-5.2–0.3)
*Age (years)*		
· 20–30		Reference
· 31–40		-6.0* (-10.2– -1.7)
· 41–50		-7.1* (-11.4– -2.8)
· 51–66		-8.1* (-12.7– -3.5)
*Income*		
· Low		Reference
· Middle		0.9 (-2.0–3.8)
· High		3.0 (-0.8–6.8)
*Marital status*		
· Married/cohabiting		Reference
· Single		3.5* (0.8–6.2)
*Time of year*		
· January-March		Reference
· April-June		0.1 (-2.3–2.5)
· July-September		-0.8 (-4.3–2.8)
· October-December		-1.7 (-4.5–1.0)

^1^Univariate linear regression.

^2^Multiple linear regression including all variables and adjusted for accelerometer wearing time in min/day.

*P < 0.05.

Numbers represent regression coefficients (with 95% confidence intervals) in minutes/day, n = 2,037.

### Meeting physical activity recommendations

The crude logistic regression model shows that having ≥4 exercise facilities within the buffer zone was associated with 70% higher odds of meeting the recommendations compared to having no exercise facilities within the buffer zone (OR = 1.70, CI = 1.39-2.08) (Table 3, model A). This difference remained significant after adjustment for sex, age, income, marital status and time of year (OR = 1.69, CI = 1.39-2.05) (Table 3, model B). None of the explanatory variables modified the association between availability of exercise facilities and meeting the physical activity recommendations.

**Table 3:** Logistic regression analysis of predictors of meeting physical activity recommendations

	**Model A**^**1**^	**Model B**^**2**^
*Availability of exercise facilities*		
· 0	Reference	Reference
· 1-3	1.06 (0.86–1.31)	1.07 (0.86–1.33)
· ≥4	1.70* (1.39–2.08)	1.69* (1.39–2.05)
*Gender*		
· Male		Reference
· Female		1.04 (0.86–1.26)
*Age (years)*		
· 20–30		Reference
· 31–40		0.78 (0.56–1.07)
· 41–50		0.88 (0.66–1.18)
· 51–66		1.09 (0.83–1.43)
*Income*		
· Low		Reference
· Middle		1.18 (0.92–1.50)
· High		1.08 (0.79–1.48)
*Marital status*		
· Married/cohabiting		Reference
· Single		1.05 (0.87–1.26)
*Time of year*		
· January-March		Reference
· April-June		1.00 (0.82–1.24)
· July-September		0.90 (0.66–1.23)
· October-December		0.82 (0.65-1.03)

^1^Univariate logistic regression.

^2^Multiple logistic regression including all variables.

Goodness of fit indices for model B: Hosmer-Lemenshow = 0.27.

*P < 0.05.

Numbers represent odds ratios (with 95% confidence intervals), n = 2,037.

## Discussion

The main findings of this study were that participants with four or more exercise facilities within 1,000-meter road network buffer zones surrounding their residences spent more time in objectively assessed moderate to vigorous physical activity, and were more likely to meet the physical activity recommendations, compared to participants with no exercise facilities within their buffer zones. This association was independent of sex, age, income, marital status and time of year.

Our findings are in accordance with the results of a previous study, which showed a significant association between objectively assessed density of exercise facilities within circular buffer zones and self-reported frequency of exercise [18]. Another study from the U.S. that investigated the association between density of exercise facilities within circular buffer zones of different sizes and a range of self-reported physical activities [13] reported similar results, although the association for the smallest buffer zones (radius 0.5 miles/805 meters) was not statistically significant. In contrast, a Spanish study found no association between numbers of exercise facilities per 10,000 inhabitants and self-reported physical activity [17]. That study measured, however, the availability of exercise facilities at the province level, and the large geographic areas used may explain the lack of association. A further study from the U.S. found no association between objectively assessed availability of exercise facilities and leisure time physical activity, as assessed using the International Physical Activity Questionnaire [34]. That study was based on relatively small circular buffer zones (radius 400 meters) and a dichotomized measure of availability of exercise facilities (yes/no).

In contrast to some previous findings [13,14], none of the socio-demographic variables included in this study (sex, age, income or marital status) modified the association between availability of exercise facilities and physical activity. In a Swedish urban setting, where differences in socioeconomic status may be less pronounced than in, for example, the U.S., individuals with different incomes seem to benefit to the same extent from exercise facilities.

Several studies have reported seasonal differences in physical activity, with higher levels of physical activity during spring and summer and a decline in activity during the colder months [20-22]. A review of the effect of season on physical activity from 2007 concluded that availability of exercise facilities could increase the opportunities to be physically active all year round in cold and wet climates [23]. However, we found no significant interaction between time of year and availability of exercise facilities in any of our analyses, suggesting that availability of exercise facilities is of equal importance for physical activity throughout the year.

The present study has some limitations that should be considered. It is a cross-sectional study and causality cannot therefore be determined. In addition, there may be unmeasured confounders for which we did not control for in the present study (i.e., residual confounding may exist). We cannot exclude the possibility that gyms and other exercise facilities may be established in neighborhoods where physically active people live, or that people who like to exercise move to neighborhoods with good availability of exercise facilities. This, together with the fact that our sample was recruited from a large urban region, may to some extent affect the generalizability of our results. It is also important to recognize that the physical activity recommendations are based on evidence from studies of self-reported physical activity and health outcomes. It is possible that misclassification occurred when assessing by accelerometry whether the physical activity recommendations were met. Another limitation is that we only measured the availability of exercise facilities around participants’ residences and not around their workplaces or their route to and from work, where they may spend a considerable amount of time [35,36]. Accelerometers may also underestimate the intensity of some physical activities performed at exercise facilities (e.g. resistance training, spinning and swimming) due to lack of mid-bodily movement and the device being non-water resistant. Compared to another population-based Swedish sample [4], our sample spent more time in moderate to vigorous physical activity (median time 42 versus 31 min/day). The other study was conducted in 2001 and its sample also included rural participants. In contrast, our sample was exclusively urban and was recruited in the capital of Sweden. However, our non-response analysis showed small or no differences in socio-demographic factors between participants and non-participants, which means that any selection bias was most likely non-differential.

The present study also has several strengths. We were able to use detailed road network data including not only roads, but also cycle paths and footpaths. There were considerable differences when visually comparing the road network alone and the road network combined with cycle paths and footpaths. The use of these detailed network data to produce line-based buffer zones around participants’ residences likely gave a good picture of the areas that are actually accessible to participants. By using objective data on availability of exercise facilities we were able to exclude the possibility of same-source bias (i.e., physically active persons reporting a higher availability of exercise facilities compared to their less active counterparts). Furthermore, accelerometers, unlike self-report, do not suffer from bias due to social desirability and recall problems [37], although it is possible that accelerometers may create some reactivity to wearing the device. However, any such bias is most likely non-differential, i.e., equal in all types of neighborhoods.

The association between availability of exercise facilities and physical activity that was identified in this study could be explained by a number of possible mechanisms. Having a large number of exercise facilities near one’s home may increase the chance of finding a mode of exercise that is attractive in terms of type of activity, cost and social atmosphere. This may explain why participants with ≥4 exercise facilities within their buffer zones were more physically active compared to those with no facilities, while participants with 1-3 facilities were not. The mere presence of exercise facilities could, by putting physical activity in the minds of passers-by, also increase the overall levels of physical activity and not just exercise performed at these facilities. In agreement with this hypothesis, Sallis et al. showed that the presence of exercise facilities close to the individuals’ homes did not seem to be associated with participation in the specific activities offered at those facilities, but rather with an increased overall exercise frequency [18].

## Conclusions

Our results show that objectively measured availability of exercise facilities is associated with accelerometer-assessed time spent in moderate to vigorous physical activity and the odds of meeting recommended levels of physical activity. Time of year had no modifying effect on these associations. Neighborhoods may be a logical and potentially significant venue for policy interventions aimed at increasing physical activity in the overall population as they have the potential to affect many people over long periods of time. In future studies, we suggest researchers to improve causal inferences by performing longitudinal studies and assess the availability of exercise facilities around people’s workplaces. Future studies are also encouraged to assess location-specific physical activity to discriminate physical activity performed within the neighborhood from that performed outside the neighborhood.

## Competing interests

None of the authors has any conflicts of interest to declare.

## Authors′ contributions

All authors contributed to the conception and design of the study. UE and KS contributed to the acquisition of data. UE performed the statistical analysis and all authors contributed to the interpretation of data. All authors contributed to revision of the manuscript for important intellectual content and approved the final version.

## Pre-publication history

The pre-publication history for this paper can be accessed here:

http://www.biomedcentral.com/1471-2458/12/607/prepub

## References

[B1] Bauman AE Updating the evidence that physical activity is good for health: an epidemiological review 2000-2003 J Sci Med Sport 2004 7 6 19 15214597 10.1016/s1440-2440(04)80273-1

[B2] Kesaniemi YK Danforth E Jensen MD Kopelman PG Lefebvre P Reeder BA Dose-response issues concerning physical activity and health: an evidence-based symposium Med Sci Sports Exerc 2001 33 S351 S358 10.1097/00005768-200106001-00003 11427759

[B3] Sundquist K Qvist J Johansson SE Sundquist J The long-term effect of physical activity on incidence of coronary heart disease: a 12-year follow-up study Prev Med 2005 41 219 225 10.1016/j.ypmed.2004.09.043 15917014

[B4] Hagstromer M Oja P Sjostrom M Physical activity and inactivity in an adult population assessed by accelerometry Med Sci Sports Exerc 2007 39 1502 1508 10.1249/mss.0b013e3180a76de5 17805081

[B5] Troiano RP Berrigan D Dodd KW Masse LC Tilert T McDowell M Physical activity in the United States measured by accelerometer Med Sci Sports Exerc 2008 40 181 188 18091006 10.1249/mss.0b013e31815a51b3

[B6] WHO Global health risks: mortality and burden of disease attributable to selected major risks 2009 Geneva: World Health Organization

[B7] Hillsdon M Foster C Thorogood M Interventions for promoting physical activity *Cochrane Db Syst Rev* 2005, **25**. 10.1002/14651858.CD003180.pub2 PMC4164373 15674903

[B8] Muller-Riemenschneider F Reinhold T Nocon M Willich SN Long-term effectiveness of interventions promoting physical activity: a systematic review Prev Med 2008 47 354 368 10.1016/j.ypmed.2008.07.006 18675845

[B9] Giles-Corti B Timperio A Bull F Pikora T Understanding physical activity environmental correlates: increased specificity for ecological models Exerc Sport Sci Rev 2005 33 175 181 10.1097/00003677-200510000-00005 16239834

[B10] Sallis JF Cervero RB Ascher W Henderson KA Kraft MK Kerr J An ecological approach to creating active living communities Annu Rev Public Health 2006 27 297 322 10.1146/annurev.publhealth.27.021405.102100 16533119

[B11] Spence JC Lee RE Toward a comprehensive model of physical activity Psychol Sport Exerc 2003 4 7 24 10.1016/S1469-0292(02)00014-6

[B12] Saelens BE Handy SL Built environment correlates of walking: a review Med Sci Sports Exerc 2008 40 S550 S566 10.1249/MSS.0b013e31817c67a4 18562973 PMC2921187

[B13] Diez Roux AV Evenson KR McGinn AP Brown DG Moore L Brines S Jacobs DR Availability of recreational resources and physical activity in adults Am J Public Health 2007 97 493 499 10.2105/AJPH.2006.087734 17267710 PMC1805019

[B14] Lee RE Cubbin C Winkleby M Contribution of neighbourhood socioeconomic status and physical activity resources to physical activity among women J Epidemiol Community Health 2007 61 882 890 10.1136/jech.2006.054098 17873224 PMC2652966

[B15] Sallis JF Saelens BE Assessment of physical activity by self-report: status, limitations, and future directions Res Q Exerc Sport 2000 71 S1 S14 10925819

[B16] Ball K Timperio A Salmon J Giles-Corti B Roberts R Crawford D Personal, social and environmental determinants of educational inequalities in walking: a multilevel study J Epidemiol Community Health 2007 61 108 114 10.1136/jech.2006.048520 17234868 PMC2465645

[B17] Pascual C Regidor E Martinez D Elisa Calle M Dominguez V Socioeconomic environment, availability of sports facilities, and jogging, swimming and gym use Health Place 2009 15 553 561 10.1016/j.healthplace.2008.08.007 18986825

[B18] Sallis JF Hovell MF Hofstetter CR Elder JP Hackley M Caspersen CJ Powell KE Distance between homes and exercise facilities related to frequency of exercise among San Diego residents Public Health Rep 1990 105 179 185 2108465 PMC1580056

[B19] Oliver LN Schuurman N Hall AW Comparing circular and network buffers to examine the influence of land use on walking for leisure and errands Int J Health Geogr 2007 6 41 10.1186/1476-072X-6-41 17883870 PMC2034381

[B20] Matthews CE Freedson PS Hebert JR Stanek EJ Merriam PA Rosal MC Ebbeling CB Ockene IS Seasonal variation in household, occupational, and leisure time physical activity: longitudinal analyses from the seasonal variation of blood cholesterol study Am J Epidemiol 2001 153 172 183 10.1093/aje/153.2.172 11159163

[B21] Newman MA Pettee KK Storti KL Richardson CR Kuller LH Kriska AM Monthly variation in physical activity levels in postmenopausal women Med Sci Sports Exerc 2009 41 322 327 19127194 10.1249/MSS.0b013e3181864c05 PMC3880933

[B22] Pivarnik JM Reeves MJ Rafferty AP Seasonal variation in adult leisure-time physical activity Med Sci Sports Exerc 2003 35 1004 1008 10.1249/01.MSS.0000069747.55950.B1 12783049

[B23] Tucker P Gilliland J The effect of season and weather on physical activity: A systematic review Public Health 2007 121 909 922 10.1016/j.puhe.2007.04.009 17920646

[B24] Sundquist K Eriksson U Kawakami N Skog L Ohlsson H Arvidsson D Neighborhood walkability, physical activity, and walking behavior: The Swedish Neighborhood and Physical Activity (SNAP) study Soc Sci Med 2011 72 1266 1273 10.1016/j.socscimed.2011.03.004 21470735

[B25] Frank LD Sallis JF Conway TL Chapman JE Saelens BE Bachman W Many pathways from land use to health - Associations between neighborhood walkability and active transportation, body mass index, and air quality J Am Plann Assoc 2006 72 75 87 10.1080/01944360608976725

[B26] Lee C Moudon AV Correlates of walking for transportation or recreation purposes J Phys Act Health 2006 3 77 10.1123/jpah.3.s1.s77 28834524

[B27] Yngve A Nilsson A Sjostrom M Ekelund U Effect of monitor placement and of activity setting on the MTI accelerometer output Med Sci Sports Exerc 2003 35 320 326 10.1249/01.MSS.0000048829.75758.A0 12569223

[B28] Matthews CE Ainsworth BE Thompson RW Bassett DR Sources of variance in daily physical activity levels as measured by an accelerometer Med Sci Sports Exerc 2002 34 1376 1381 10.1097/00005768-200208000-00021 12165695

[B29] Freedson PS Melanson E Sirard J Calibration of the Computer Science and Applications. Inc. accelerometer Med Sci Sports Exerc 1998 30 777 781 10.1097/00005768-199805000-00021 9588623

[B30] WHO Global Recomendations on Physical Activity for health 2010 Geneva: World Health Organization 26180873

[B31] Masse LC Fuemmeler BF Anderson CB Matthews CE Trost SG Catellier DJ Treuth M Accelerometer data reduction: a comparison of four reduction algorithms on select outcome variables Med Sci Sports Exerc 2005 37 S544 S554 10.1249/01.mss.0000185674.09066.8a 16294117

[B32] Cerin E Leslie E Owen N Explaining socio-economic status differences in walking for transport: an ecological analysis of individual, social and environmental factors Soc Sci Med 2009 68 1013 1020 10.1016/j.socscimed.2009.01.008 19193480

[B33] Hosmer DW Lemeshow S Applied logistic regression 2000 New York: Wiley-Interscience

[B34] Hoehner CM Brennan Ramirez LK Elliott MB Handy SL Brownson RC Perceived and objective environmental measures and physical activity among urban adults Am J Prev Med 2005 28 105 116 10.1016/j.amepre.2004.10.023 15694518

[B35] Rodriguez DA Brown AL Troped PJ Portable global positioning units to complement accelerometry-based physical activity monitors Med Sci Sports Exerc 2005 37 S572 S581 10.1249/01.mss.0000185297.72328.ce 16294120

[B36] Troped PJ Wilson JS Matthews CE Cromley EK Melly SJ The built environment and location-based physical activity Am J Prev Med 2010 38 429 438 10.1016/j.amepre.2009.12.032 20307812 PMC3568665

[B37] Ward DS Evenson KR Vaughn A Rodgers AB Troiano RP Accelerometer use in physical activity: best practices and research recommendations Med Sci Sports Exerc 2005 37 S582 S588 10.1249/01.mss.0000185292.71933.91 16294121

